# Twenty years on from smoke-free legislation in Scotland: A secondary analysis of the Scottish Health Survey dataset (1998–2024) examining changes in household smoking rules, and salivary cotinine concentrations among non-smokers

**DOI:** 10.18332/tid/219319

**Published:** 2026-03-25

**Authors:** Sean Semple, Catherine Best, Emma Riches, Lynne Morton, Garth Reid, Rebecca Howell, Rachel O’Donnell

**Affiliations:** 1 Institute for Social Marketing and Health, Faculty of Health Sciences and Sport, University of Stirling, Stirling, Scotland; 2 Population Health and Wellbeing, Public Health Scotland, Edinburgh, Scotland

**Keywords:** secondhand smoke, salivary cotinine, smoke-free, public health, trends

## Abstract

**INTRODUCTION:**

Scotland introduced comprehensive smoke-free legislation covering most enclosed public spaces in 2006. Twenty years on, this study examines changes in markers of population level exposure to secondhand tobacco smoke (SHS).

**METHODS:**

A secondary analysis of Scottish Health Survey data between 1998 and 2024 to examine trends in population exposure to SHS and household rules about smoking indoors. The proportions of non-smoking adults who had measurable cotinine in their saliva were calculated for the period 1998–2024. The geometric mean (GM) concentrations of cotinine levels were calculated using Tobit regression. Data from 2012–2024 on self-reported smoking rules for the home were analyzed.

**RESULTS:**

Salivary cotinine expressed as a GM fell from 0.464 ng/mL (95% CI: 0.444–0.485) in 1998 to 0.020 ng/mL (95% CI: 0.015–0.028) in 2024: a reduction of 95.7%. The percentage of non-smoking adults who had no measurable cotinine in their saliva increased by six-fold between 1998 (12.5%) and 2024 (77.6%). Most of the change occurred in the immediate aftermath of smoke-free legislation, with both metrics of population exposure to SHS demonstrating little evidence of change between 2011 and 2024. The proportion of households that are smoke-free has increased from 75.2% in 2012 to 90.2% in 2024 but is now ten times more common in the most deprived areas compared to the least deprived.

**CONCLUSIONS:**

Scotland has sustained large reductions in SHS exposure since smoke-free legislation was introduced twenty years ago in 2006. However, progress evident in the years between 2006 and 2011 has not been maintained: there are still nearly one-quarter of non-smoking adults having measurable exposure to SHS on any given day. Smoking in the home has also reduced, but the level of inequality of this measure has doubled between 2012 and 2024. Public health interventions should consider the remaining workplace and home settings where people experience exposure to SHS.

## INTRODUCTION

As a result of the World Health Organization (WHO) Framework Convention on Tobacco Control (FCTC), global progress has been made in protecting non-smokers from secondhand tobacco smoke (SHS) in workplaces and enclosed public spaces^1^. The Republic of Ireland led the way in introducing national-level smoke-free enclosed public spaces to protect non-smokers from the harms of SHS in 2003^2^, and many countries followed soon after. Today, 2.1 billion people across 74 countries benefit from comprehensive smoke-free policies in indoor public places, workplaces, and on public transport^3^.

Scotland introduced comprehensive legislation banning smoking in enclosed and partially enclosed public spaces on 26 March 2006^4^. Across the UK, England, Wales and Northern Ireland followed with near identical measures at various points in 2007. Smoke-free measures have been widely demonstrated to reduce exposure to SHS^5,6^ and to have major public health benefits helping to improve child^7^ and adult health^8^, and reduce exacerbations of existing conditions^9^.

In the twenty years since smoke-free legislation came into force in Scotland, a range of other policies and measures to protect specific population groups have been introduced. These have included: a governmental target to reduce the proportion of children exposed to SHS in their own home, backed by a national mass media campaign (‘Take it Right Outside’) (2014)^10^; legislation to prohibit smoking in vehicles carrying children (2016)^11^; smoke-free prisons in Scotland (2018)^12^; and legislation to prohibit smoking within 15 m of NHS hospital buildings (2022)^13^. Despite these measures there is evidence of continuing exposure to SHS both in the workplace and in home settings. A job exposure matrix in 2020 concluded that at least 1.04 million workers in the UK are likely to be exposed to SHS while performing their job^14^, with many workers exposed in outdoor hospitality settings where the 2006 regulations do not apply or when working in or visiting private homes. A 2025 survey found 20% of workers in the UK self-reported exposure to SHS within the last three months^15^, with those working in the transport and hospitality sectors reporting exposure most. Data on home healthcare workers have also demonstrated that this group is at particular risk of SHS during visits to domestic settings where smoking takes place^16^.

Although there is strong evidence that smoke-free interventions are cost-effective^17^, there is no requirement to tackle SHS exposure in the home setting in Article 8 of the WHO FCTC^18^. The absence of guidance within Article 8 about the need to protect non-smokers from SHS in the home is stark, given the home setting is where many non-smokers experience exposure now that most enclosed and partially enclosed public spaces are smoke-free in those countries with well-enforced smoke-free legislation^19^. Workplace exposure is often of short duration and, if outdoors, at low concentrations. Data show that concentrations of SHS in homes where people smoke are often much higher than those previously found in work settings^20^.

Previous analysis^21^ of longitudinal, population survey data in Scotland demonstrated a reduction in SHS exposure among non-smokers: the mean value of cotinine decreasing by over 97% between 1998 (prior to the legislation) and 2016 (ten years after implementation). The analysis also showed that the proportion of non-smoking adults with undetectable levels of cotinine increased from 12.5% in 1998 to as high as 81.6% by 2016.

Providing policymakers and other stakeholders with continuing analysis of non-smokers’ exposure to SHS is one of the requirements of Article 8 in the WHO FCTC. Given that the 20th anniversary of Scotland’s smoke-free measures is imminent on 26 March 2026 and that Scotland has a target of reducing smoking prevalence to <5% by 2034^22^, there is a need to understand how population exposure to SHS has continued to change and the role that smoke-free spaces can play in Scotland’s tobacco endgame. This study builds on a previous analysis carried out on the Scottish Health Survey to 2016^21^, extending the longitudinal data on salivary cotinine to 2024, and adds new analysis of self-reported data on household smoking rules gathered since 2012. The analysis seeks to: determine if previously reported progress in protecting non-smokers from SHS has been sustained; how social norms are changing in terms of smoking rules in the home; and consider additional measures that Scotland, and other countries, could implement as part of the tobacco endgame in relation to SHS.

## METHODS

A secondary analysis of annual Scottish Health Survey data between 1998 and 2024 was performed to examine trends in population exposure to SHS and household rules about smoking indoors. Scottish Health Survey data from 1998 to 2016 were acquired from the UK Data Service^23^ and analyzed previously^21^. Additional anonymized data from 2017–2022 were accessed using the same methods, with the 2023 and 2024 anonymized datasets provided directly from Scottish Government statistics via a Public Benefit and Privacy Panel data request. The Scottish Health Survey is a nationally representative sample of the population living in households in Scotland – full details of sample selection and data collection methods are available in each annual report^24^.

Saliva samples were first gathered from a sub-sample of health survey participants in 1998, again in 2003, and were then gathered annually in the survey since 2008 – with the exception of 2020 and 2021 due to COVID-19 restrictions. The method of saliva sample collection has remained the same during the entire period reported here. The method of analysis of saliva samples used since the 2009 Scottish Health Survey is high performance liquid chromatography coupled to tandem mass spectrometry with multiple reaction monitoring (LC-MS/MS), replacing the gas chromatography nitrogen phosphorous detection (GC-NPD) method used in the 1998, 2003 and 2008 surveys. The sample preparation prior to LC-MS/MS was liquid/liquid extraction^25^. The Limit of Detection (LOD) has remained the same during the entire period reported in this study.

To align with the previous analysis^21^, data were restricted to those who were aged ≥16 years and had a valid cotinine sample with a measure below 12 ng/mL, the cut-point for validated non-smoking adults^26^ (n=17830; ranging from 281 non-smoking adults in 2022 to 3738 in 1998). The proportion of valid samples below 0.1 ng/mL, the LOD for salivary cotinine, was calculated for each annual survey. Tobit regression of log-transformed concentrations with the LOD as the lower limit was used to determine the geometric mean (GM) and 95% confidence interval (CI) estimates separately for each year. This method is recommended for use in datasets containing a substantial proportion of values below the LOD^27^. Data were extracted from the original SPSS files downloaded from the UK Data Service website or provided from the Scottish Government, and analysis was performed in Microsoft Excel and Stata BE version 17 (Stata Corporation, College Station, TX, USA^28^). Absolute change in GM cotinine and the proportion of non-smokers without detectable cotinine was calculated for the period previously analyzed (1998–2016) and the updated time period 2017–2024. The trend during the period (2008–2012) directly after smoke-free legislation was implemented in Scotland in 2006 was compared to the trend in more recent years (2013–2024) by fitting a segmented linear regression line by time period to a Tobit regression model including data from all years.

A question on household smoking rules was first introduced to the survey in 2012. ‘What best describes the smoking rules in this house/flat?’ was asked of all households each year and provided the following options: People can smoke anywhere inside this house/flat; People can only smoke in certain areas or rooms inside this house/flat; People can only smoke in outdoor areas; People cannot smoke indoors or in outdoor areas of this house/flat. Responses to the final two of these statements were categorized as a ‘smoke-free home’. Data were not available for 2020 due to COVID-19 and data from 2021 were excluded as these were collected by telephone interview rather than the traditional home visit used in all other surveys. A total of 70729 valid responses were available across the other years ranging from 5296 in 2017 to 7132 in 2023. Responses were also analyzed by the Scottish Index of Multiple Deprivation (SIMD)^29^ in both 2012 and 2024 to calculate the relative change by SIMD quintile and determine any potential widening or narrowing of inequality in this important measure of SHS exposure.

## RESULTS

**Figure 1:**
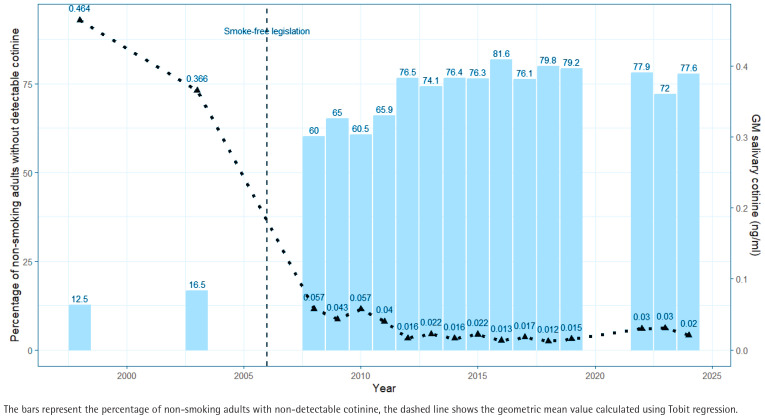
Secondary analysis of Scottish Health Survey salivary cotinine data from non-smoking adults, 1998–2024 (N=17830)

Figure 1 provides details of the salivary cotinine concentrations measured in each year from 1998 to 2024. The population geometric mean (GM) cotinine concentration, calculated by Tobit regression, is presented, together with details of the proportion of non-smokers who have no measurable level of cotinine on the day of sample collection. The overall change in salivary cotinine concentrations between 1998 and 2024 is substantial, decreasing from 0.464 ng/mL (95% CI: 0.444–0.485) in 1998 to 0.020 ng/mL (95% CI: 0.015–0.028) in 2024, a reduction of 95.7% demonstrating the continuing success of smoke-free legislation introduced in 2006.

Comparison of the change in both salivary cotinine concentrations, and the proportion of non-smokers with undetectable levels, since the last previous analysis carried out on data to 2016 is provided in Table 1. This analysis shows that improvements in protection of non-smokers from SHS increased steadily most years until 2016, but that this trend of greater population protection has not continued in recent years: both metrics appearing to flatline or show indications of increasing exposure to nicotine in the past decade.

**Table 1:** Changes in geometric mean salivary cotinine concentrations and the proportion of non-smoking adults with undetectable cotinine levels in Scotland, based on Scottish Health Survey data, 1998–2024 (N=17830)

*Period*	*Change in GM* *salivary cotinine* *(ng/mL)*	*Change in proportion of* *non-smokers with no* *detectable cotinine*
1998–2016	-0.451 (-97.2%)	69 %
2016–2024	0.007 (55.4%)	-4 %

GM: geometric mean.

The segmented Tobit regression analysis confirms this interpretation. Between 1998 and 2005, the gradient of the slope was negative and statistically significant (-0.0497 log salivary cotinine, 95% CI: -0.065 – -0.034) and the same in 2006–2012 (-0.118 log salivary cotinine, 95% CI: -0.164 – -0.073). However, after 2012 the slope is no longer statistically significant (-0.007 log salivary cotinine, 95% CI: -0.020–0.006) indicating no further decrease over time.

**Figure 2:**
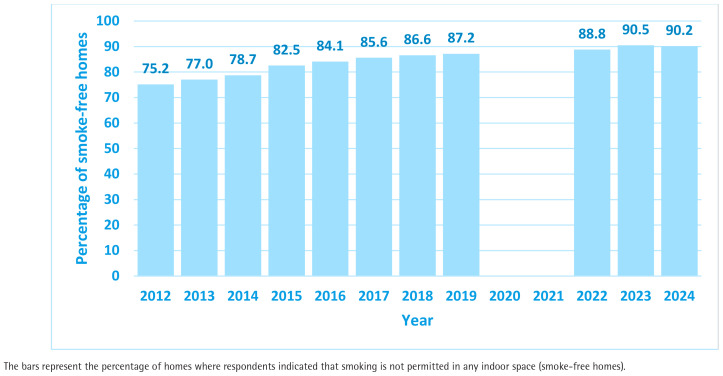
Secondary analysis of Scottish Health Survey percentage of smoke-free homes, 2012–2024 (N=70729)

Figure 2 provides details of the proportion of homes in Scotland that have self-reported rules that prohibit smoking within the indoor space (i.e. are classified as ‘smoke-free’). The proportion has risen steadily each year from 75.2% in 2012, when this question was first asked in the Scottish Health Survey, to a peak of 90.5% in 2023 before dropping very slightly to 90.2% in the latest data in 2024. Using data on the total number of households in Scotland for 2024^30^, this analysis suggests that approximately 380000 homes in Scotland have shifted to become ‘smoke-free’ in the past 12 years.

**Figure 3:**
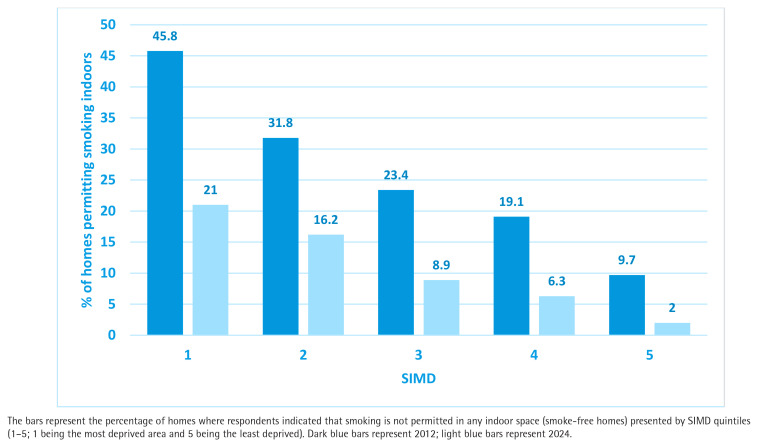
Secondary analysis of Scottish Health Survey percentage of homes where smoking is permitted indoors by Scottish Index of Multiple Deprivation quintiles in 2012 (N=6588) and 2024 (N=6573)

Figure 3 provides detail of the proportion of smoking permitted homes by SIMD quintile in both 2012 and 2024, and demonstrates that while all socioeconomic categories have seen improvement, the greatest relative improvement has occurred in the most affluent homes (SIMD 5). Homes in low-income areas (SIMD1) have experienced a reduction in smoking-permitted homes from 45.8% in 2012 to 21.0% in 2024; while SIMD 5 has seen a fall from 9.7% in 2012 to 2.0% in 2024. This demonstrates a widening of exposure inequality from a ratio of 4.72 times in 2012 to 10.5 times in 2024: a more than doubling in the inequality of risk of living in a smoking-permitted home between the most and least deprived households in Scotland.

## DISCUSSION

Previous analysis demonstrated that smoke-free legislation produced major reductions in population level salivary cotinine in the decade after implementation^21^. This work extends this analysis through to 2024 and shows that reductions are sustained and continuing nearly 20 years on from this global-leading public health measure. Evidence of the sustained reduction appears both in the population-level measure of non-smokers’ mean salivary cotinine and in the total proportion of non-smokers who have undetectable levels of cotinine. The health benefits that continue to accrue from the protection from SHS exposure that workers and the public now experience are likely to be substantial and sustained, demonstrating the ongoing public health success of legislation introduced 20 years ago.

While these recognized improvements in protection are welcome, these data also suggest that nearly 1 in 4 non-smoking adults in Scotland continues to breathe SHS on any given day. The results show that 22.4% of non-smoking adults have measurable levels of cotinine in 2024. The population-level GM value of salivary cotinine is, however, much lower now than in the years preceding or immediately post-implementation, suggesting that most of those who continue to be exposed are receiving a much lower inhaled dose of SHS than twenty years ago.

Vaping and e-cigarette use has increased markedly in Scotland over the past 20 years with latest prevalence figures indicating 12% of adults use these devices^31^. Those who vape or use other nicotine products, are likely to have salivary cotinine concentrations >12 ng/mL^32^ and so will not be included in the non-smoking sample in our analysis. While there is some potential for nicotine intake through exposure to secondhand aerosol from vaping, data from studies that have measured fine particulate matter (PM2.5) concentrations in homes where vaping takes place are broadly similar to smoke-free homes^33^. Salivary cotinine concentrations in people who live with those who vape suggests that nicotine intake from secondhand aerosol is much lower than that from cigarette SHS^34^. As a result, we are confident that salivary cotinine, as reported here, is a robust indicator of SHS exposure and unlikely to have been influenced by the increased prevalence of vaping behavior.

Recent work has suggested that 1 in 5 workers in the UK continues to report exposure to SHS^15^. This aligns well with a job-exposure matrix suggesting there are a total of 10.4 million workers in the UK employed in jobs (representing 22.6% of jobs held) identified as likely to experience some degree of exposure to SHS at work^14^. That study estimated that approximately 10% of those workers (1.04 million) are likely to experience SHS exposure on any given day^14^. The Tobacco and Vapes bill currently progressing through the UK parliament (at time of writing it is at the committee stage in the House of Lords) will provide the powers to extend smoke-free spaces to offer additional protection to workers^35^. These powers offer an opportunity for governments across the UK to consider how best to protect workforces that continue to be exposed to SHS.

The data on household smoking rules point to marked shifts in the social norms relating to the acceptability of smoking in the home. In the space of 12 years, the fraction of homes permitting smoking indoors has more than halved from 24.8% to 9.8%; this societal change is likely to have been generated by two factors: 1) the reduced prevalence of smoking with overall adult smoking rates in Scotland falling from 25% in 2012 to 14% by 2024^24^; and 2) the indirect impact of the 2006 smoke-free regulations on social norms, attitudes and knowledge relating to the health harms of SHS, which has thus markedly decreased the acceptability of smoking in the home. The reduction in smoking-permitted homes between 2012 and 2024 has occurred across all socioeconomic categories, but the data demonstrate that benefits have been seen disproportionately in more affluent homes compared to the most deprived 20% of households. The inequalities gap associated with living in a home where smoking takes place has doubled, suggesting that greater targeting and help for smokers in SIMD 1 to create a smoke-free home is required.

Potential measures to encourage creation of smoke-free homes include: incorporating specific support strategies within national cessation frameworks like Scotland’s ‘Quit Your Way’^36^; providing people who smoke with personalized household feedback to demonstrate the impact of smoking indoors on air quality^37^; and provision of free nicotine replacement therapy (NRT) for the purposes of temporary abstinence in the home^38^. There is good evidence that creating a smoke-free home can serve as a stepping-stone to successful cessation, including for individuals who are not motivated to quit completely^39^. In addition, creating a smoke-free home is likely to be of particular benefit to children and those with existing respiratory and cardiovascular health conditions^40^.

Current data suggest Scotland is not on target to achieve the target of <5% smoking prevalence by 2034 unless additional measures are put in place^41^. Reducing exposure to SHS has a key role to play in Scotland’s tobacco endgame through helping to change societal norms about restricting smoking in more spaces and limiting opportunities to smoke. A renewed emphasis on providing protection from SHS also aligns well with the prevention focus that forms the center of Scotland’s recently launched Population Health Framework that aims to tackle the root causes of ill-health in our communities^42^. The UK Tobacco and Vapes bill seeks to introduce a rising age of sale for tobacco, with those born after 1 January 2009 unable to purchase tobacco products^35^. This measure has the potential to further shift the social norm and unacceptability of smoking in the presence of individuals who do not smoke, and may provide future improvements in the metrics considered in this study. At a European level, the recent (2024) recommendations from the 2nd Joint Action on Tobacco Control^43^ also suggest expansion of smoke- and aerosol-free environments to include a range of outdoor, indoor and semi-enclosed settings to offer better protection from SHS.

### Limitations

The study has a number of limitations. The data on household smoking rules are based on self-report and are thus subject to reporting bias. There is the potential for a greater knowledge of the harms caused by SHS and increasing stigma in relation to smoking, to cause respondents to provide the socially acceptable answer to this question. The survey is a repeated cross-sectional survey and so those taking part are unlikely to have been asked this question in previous years. Additionally, the question is placed within a wide-ranging survey covering a whole range of lifestyle and health factors including mental and physical health, alcohol use, diet and physical activity, and so respondent bias may be minimized. A second weakness lies in the fact that salivary cotinine, with a half-life of 16–20 hours, measures recent exposure to nicotine and does not reflect long-term exposure. As such the figures presented here are indicative of exposure to SHS over the past 1–2 days and so may be an under-estimate of population exposure on a weekly or less regular basis. However, the methods used to collect samples, measure and quantify exposure across the 1998–2024 period were identical and so results from each year are comparable to each other.

## CONCLUSIONS

Scotland has sustained large reductions in SHS exposure since smoke-free legislation was introduced twenty years ago in 2006. However, progress seen in the early years post-legislation has not been maintained with the most recent figures in 2024 showing a level comparable to 2012 with nearly one-quarter of non-smoking adults still having quantifiable exposure to SHS on any given day. Smoking in the home has also decreased but is now ten times more common in homes in the most deprived areas compared to the least deprived. Public health interventions should consider the remaining workplace and home settings where people still experience exposure to SHS.

## Data Availability

The data supporting this research are available from the following link: https://ukdataservice.ac.uk/
